# Tele-mentoring - a way to expand laparoscopic simulator training for medical students over large distances: a prospective randomized pilot study

**DOI:** 10.1186/s12909-023-04719-x

**Published:** 2023-10-10

**Authors:** Ninos Oussi, Emil Forsberg, Michael Dahlberg, Lars Enochsson

**Affiliations:** 1 Centre for Clinical Research, Region Sörmland, Uppsala University, Eskilstuna, Sweden; 2 Division of Urology, CLINTEC, Karolinska Institutet, Stockholm, Sweden; 3 Department of Surgical and Perioperative Sciences, Division of Surgery, Umeå University, Umeå, Sweden; 4 Department of Surgery, Sunderby Hospital, Luleå, 971 80 Sweden; 5 Division of Surgery, CLINTEC, Karolinska Institutet, Stockholm, Sweden

**Keywords:** Simulation, Laparoscopy, Training, Tele-mentoring, Sparsely populated, Outcome

## Abstract

**Background:**

Studies have shown the clinical benefits of laparoscopic simulator training. Decreasing numbers of operations by surgical residents have further increased the need for surgical simulator training. However, many surgical simulators in Sweden are often insufficiently used or not used at all. Furthermore, large geographical distances make access to curriculum-based surgical simulator training at established simulator centres difficult. The aim of this study was to evaluate whether tele-mentoring (TM) could be well tolerated and improve basic laparoscopic surgical skills of medical students 900 km away from the teacher.

**Methods:**

Twenty students completed an informed consent and a pre-experimental questionnaire. The students were randomized into two groups: (1) TM (N = 10), receiving instructor feedback via video-link and (2) control group (CG, N = 10) with lone practice. Initial warm-up occurred in the Simball Box simulator with one Rope Race task followed by five consecutive Rope Race and three Peg Picker tasks. Afterwards, all students completed a second questionnaire.

**Results:**

The whole group enjoyed the simulator training (prescore 73.3% versus postscore 89.2%, *P* < 0.0001). With TM, the simulator Rope Race overall score increased (prescore 30.8% versus postscore 43.4%; *P* = 0.004), and the distance that the laparoscopic instruments moved decreased by 40% (*P* = 0.015), indicating better precision, whereas in the CG it did not. In Peg Picker, the overall scores increased, whereas total time and distance of the instruments decreased in both groups, indicating better performance and precision.

**Conclusions:**

Simulation training was highly appreciated overall. The TM group showed better overall performance with increased precision in what we believe to be the visuospatially more demanding Rope Race tasks compared to the CG. We suggest that surgical simulator tele-mentoring over long distances could be a viable way to both motivate and increase laparoscopic basic skills training in the future.

**Supplementary Information:**

The online version contains supplementary material available at 10.1186/s12909-023-04719-x.

## Background

With the development of advanced surgical simulators, the opportunities for surgeons to undergo basic and advanced surgical skills training without putting the patient at risk have increased. Those who use surgical simulators in surgical training show proficiency in laparoscopic skills transferable to the operating room [1]. Furthermore, several prospective randomized studies have shown enhanced surgical skills development and performance in laparoscopic cholecystectomy when training with surgical simulators [2–4]. Furthermore, warming-up in a laparoscopic simulator prior to surgery has shown improved surgical outcome [5]. However, although the availability of validated surgical simulators is slowly increasing much of the initial practice is still done on patients, which constitutes a potential hazard to patient safety [6, 7]. In order to overcome the high cost of advanced medical simulators, a number of low cost simulators have entered the market in recent decades [8–10]. However, these simulators are often limited to training basic endoscopic and laparoscopic skills and rarely offer the opportunity to practice more advanced techniques [11]. Thus, although, mostly low-cost, surgical simulators are available at many hospitals, yet the absence of systematic training within a specified curricula and geographical distances to accredited simulation centres affects the training of junior surgeons in a negative way [12].

Moreover, the lack of simulator feedback is an obstacle to effective learning and something to consider when planning a simulator training curriculum. The benefits of instructor feedback for practical performances, such as surgical simulation training, has been widely acknowledged [13–15]. Intrinsic motivation is also enhanced after simulation-based team training [16], thus, student attitudes, not least of which is the motivation to train, should be considered when planning a training curriculum. Just as important is to eliminate the greatest barrier to voluntary training, i.e. lack of available free time [17]. Furthermore, a combination of different feedback techniques, such as structured video self-assessment, has been suggested to alter learning proficiency with respect to surgical skills [18]. However, instructor feedback given during laparoscopic simulator training had no influence on the retention of skills in the long-run [19]. Attempts have been made to analyse and improve the conditions by which the simulator itself can provide the diagnostic assessment of a novice´s problem areas to provide directed self-guided learning [20].

In concordance with the coronavirus 2019 (Covid-19) pandemic, homes, societies, and workplaces were enforced different restrictions [21, 22]. During the pandemic healthcare systems were decimated and surgery was withheld thus affecting the levels of patient care [23]. Subsequently, surgical training also suffered from these issues since younger surgeons were removed from surgical and OR training due to the downscaling of surgery; henceforth, a shift from traditional training models to remote learning was suggested [24–27] not least through the digitalization process [28]. Thus, several institutions, to some extent even though the pandemic has ended, have reorganized their surgical resident training to prevent a decline in practical surgical skills during training. With the new and innovative approach to the challenges that the pandemic presented, the negative impact on residency training might be reduced [29–33].

Regardless of the effect that the Covid-19 pandemic had on surgical residency training, a survey by the Fellowship Council Research Committee sent to the program directors of all surgical subspecialities in North America presented an unsatisfying number of residents who were not fully equipped for undertaking laparoscopic procedures in the OR [34].

One of the solutions provided during the pandemic was the accessibility to video-conference calls, meetings, and online education [35]. Surgical tele-mentoring has been reported for decades but the level to which it improves practical surgical skills and the clinical outcome is unclear [36]. Perhaps, one way of improving the scarce simulation training regardless of geographical distances could be by providing instructor feedback and integration of instructional media in conjunction with tele-mentoring [37].

The aim of this study was to assess whether tele-mentoring (TM) could improve the learning of basic laparoscopic skills via simulator training of medical students 900 km away from the teacher.

The primary hypothesis stated that tele-mentoring can be given over large distances and will be well received by the students.

The secondary hypothesis stated that this type of teaching can also objectively add improved outcomes in simulated basic surgical skills.

**Table 1:** Demographic data of the respective groups

		Tele-mentoring	Control group	*P*
Sex	Female	3	7	0.074
	Male	7	3	
Age (years)*		25.5 ± 2.8	25.3 ± 2.5	0.870
Dexterity	Righthanded	9	10	0.305
	Lefthanded	1	0	
Playing computer games (%) *		68.0 ± 31.7	30.9 ± 34.6	**0.023**
Approximate mobile daily screen time (hours)	0–2 h	2	1	0.402
	2–4 h	4	6	
	4–6 h	2	3	
	> 6 h	2	0	

*Values are mean ± standard deviation

*P* < 0.05 statistically significant

**Table 2:** Pre-experiment vs. post-experiment emotions

		Tele-mentoring		
	Pre- experiment	Post-experiment	*P* (matched pairs)	Mean difference
Training will be/was difficult	60.8 ± 15.2	61.7 ± 11.8	0.900	0.9
Will like/liked the training	74.8 ± 11.4	92.7 ± 7.3	**0.000**	17.9
		**Control Group**		
	**Pre- experiment**	**Post-experiment**	***P*** **(matched pairs)**	**Mean difference**
Training will be/was difficult	69.4 ± 19.5	54.7 ± 22.3	0.160	-14.7
Will like/liked the training	71.8 ± 15.3	85.6 ± 10.4	**0.001**	13.8
% (mean ± standard deviation)				

*P* < 0.05 statistically significant

## Methods

**Fig. 1:**
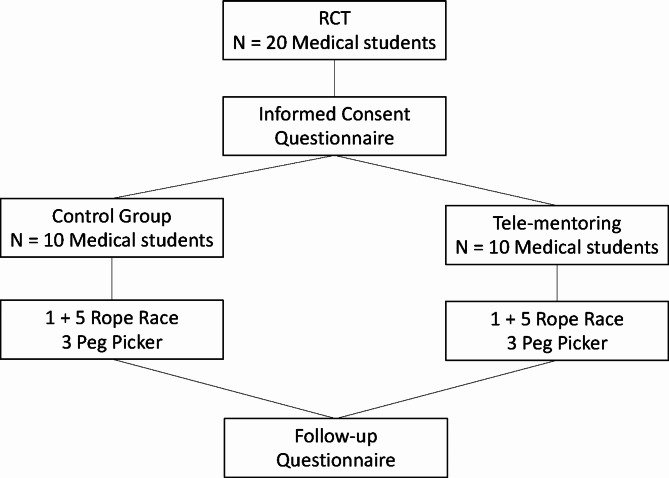
Flowchart of the study design

A cohort of 20 medical students (10 females and 10 males) with a mean age of 25.4 ± 2.6 years (mean ± standard deviation [SD]) volunteered to participate in the study. All subjects signed an informed consent and completed a questionnaire with some background factors, including experience and attitude towards simulation training and tele-mentoring, prior to the study. The subjects were medical students during their surgical semester at Umeå University, Umeå, Sweden. These students were naïve to both laparoscopy and laparoscopic simulator training. The study was conducted at Sunderby Hospital, Luleå, Sweden where the participating students did their surgical semester. The students were randomized into two equally large groups, performing basic skills training using the Simball^®^ Box laparoscopic simulator (Surgical Science Sweden AB, Gothenburg, Sweden) for which 10 subjects received tele-mentoring (TM) and the control group (CG) with 10 subjects performed a lone practice (Fig. 1). The control group performed the tasks according to the instructions given by the simulator video prior to and in conjunction with the tasks. The TM group also followed the pre-task video instructions given by the simulator. However, the TM group also received Zoom^®^ feedback from a senior instructor in Stockholm who was located 900 km from Sunderby Hospital in Luleå. The instructions to the TM group were mostly given by the remote instructor over Zoom between each individual task in order not to interfere with the procedures. Occasionally, instructions were also given during the exercises if an obvious mistake was made that led to that the student was not able to complete the task. The instructions given to the TM group were not completely standardized but in the Rope Race procedure the most common mistake was that the students grasped the end of the rope with the instrument at a wrong angle and then tried to force the rope through the loop. In the Peg Picker task, the most common mistake was that they grasped the peg with the wrong instrument. During the whole experiment a representative of the faculty, EF, was present in order to observe the performance of the subjects both in the TM as well as the CG-groups to make sure that the experiments were completed according to the initial plan of the project.

### The setup

**Fig. 2:**
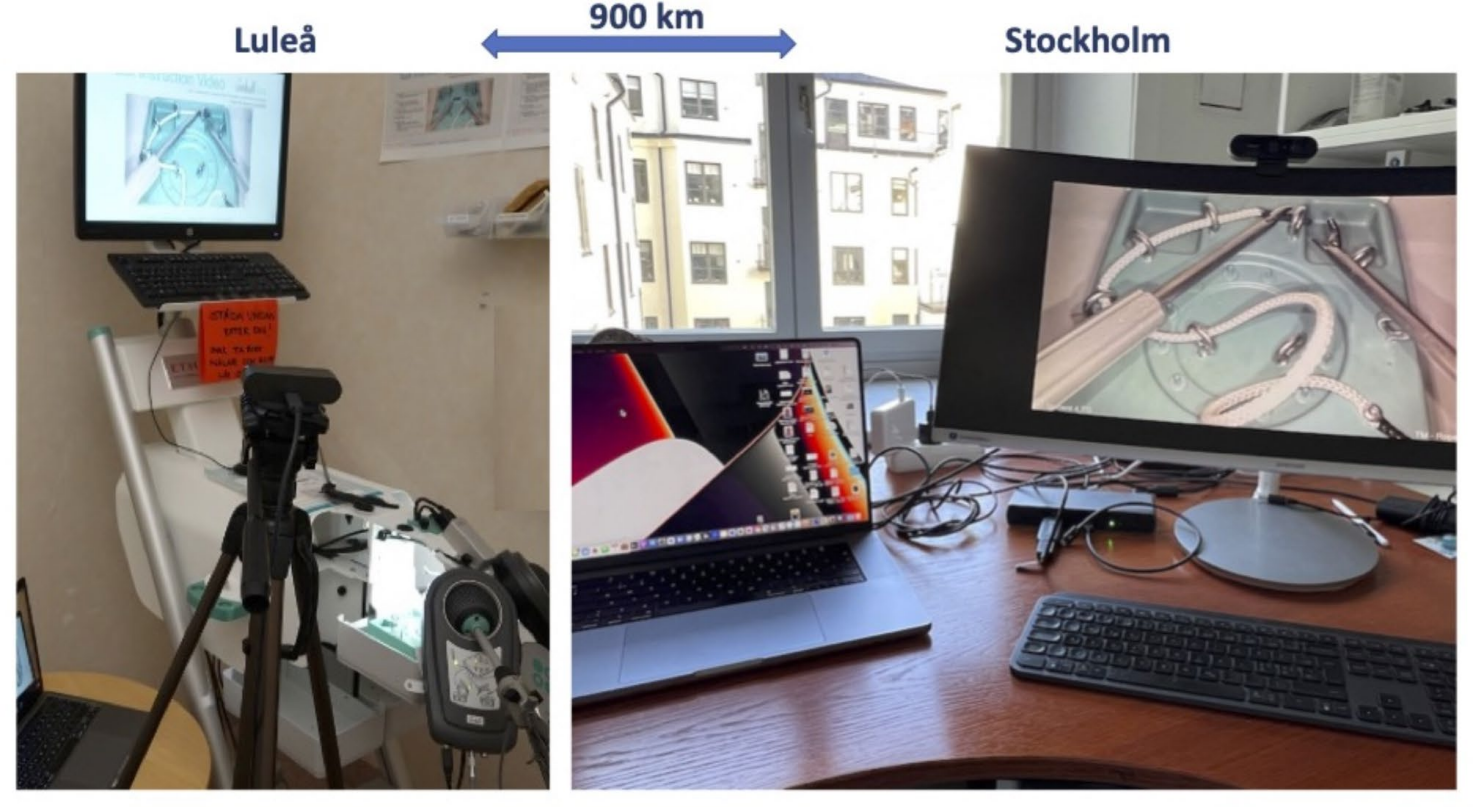
The Simball Box setup at Sunderby Hospital, Luleå (left) and the computer setup for the teacher in Stockholm (right)

At the site in Luleå, the Simball Box was attached to an ASUS^®^ laptop (Asustek, Taipei, Taiwan) with an Intel^®^ (Intel Corp, California, USA) Core i7-4510U CPU and a 13.3 Inch screen. Furthermore, a Logitech Brio^®^ Webcam (Logitech International SA, Lausanne, Switzerland) was attached to the ASUS computer. Two streaming channels over Zoom^®^ (Zoom Video Communications Inc., San Jose, California, USA) reached the instructor: (1) The picture from the Simball Box screen was directly streamed over one of the channels and (2) The Logitech Brio signal, which was focused on the hand movements, was streamed from the ASUS computer via channel nr 2 (Fig. 2).

The instructor at Stockholm was connected to Zoom via an Apple MacBook Pro^®^ 16-inch with an M1 Max processor (Apple Inc., Cupertino, California, USA) and a Logitech Brio^®^ Webcam, which was mounted on a Samsung^®^ 34 Inch Colour Display Unit (C34J791WT) (Samsung Electronics, Republic of Korea) as shown in Fig. 2.

### Experiments

**Fig. 3:**
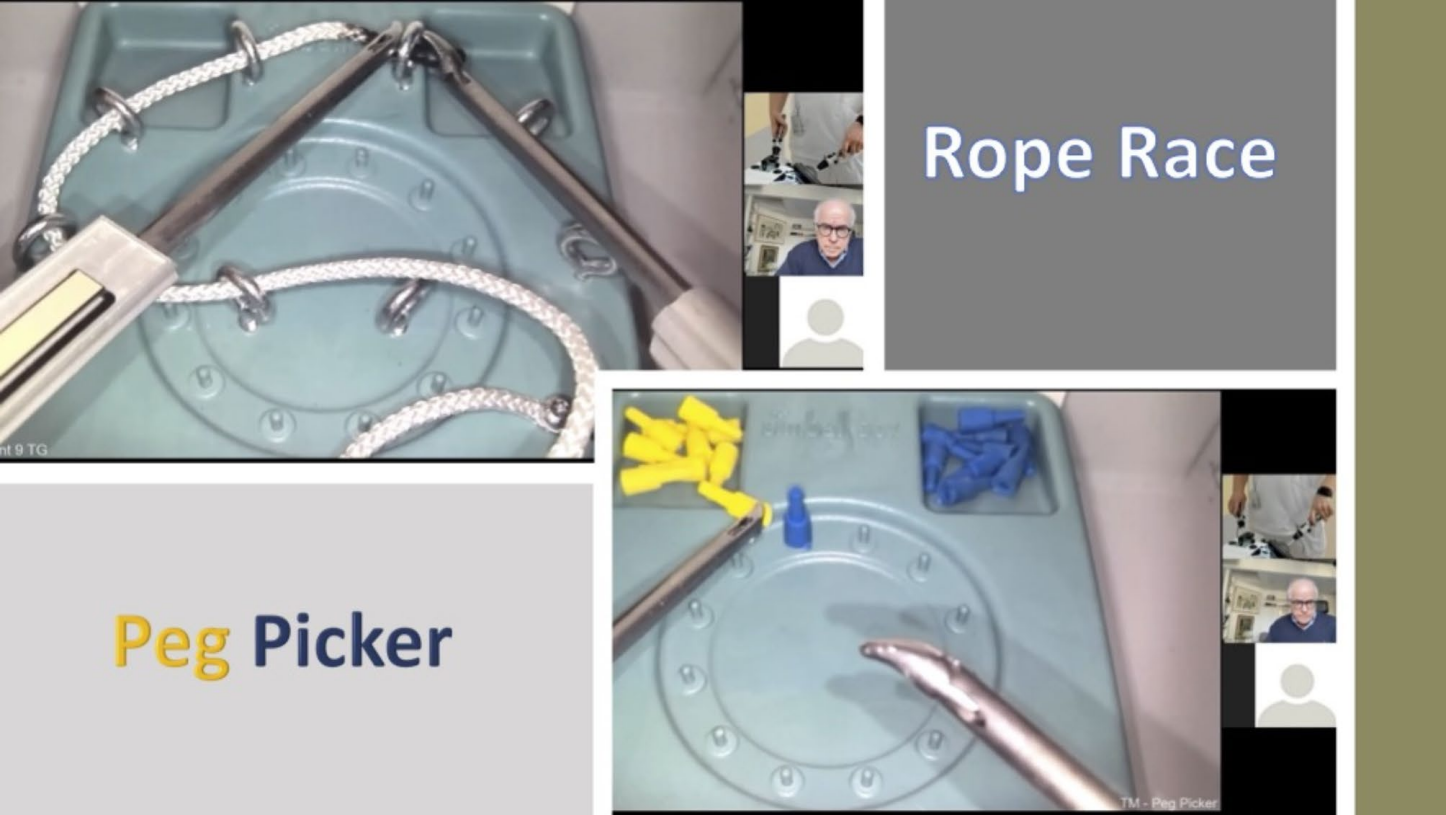
How the simulator experiments with Rope Race (upper left) and Peg Picker (lower right) are displayed for the teacher in Stockholm. The teacher seen in the picture in this figure is the corresponding author and PI of this study who has given his consent for his face to be seen in the picture

All subjects performed the basic skills tasks, “Rope Race” and “Peg Picker”, in the simulator (Fig. 3). They started with a warmup performance of one Rope Race during which the test subject threads a thin rope through eight loops placed in a circle. The placement of the loops requires the subject to grasp the rope with their surgical instruments at different angles in order to pass the rope successfully through each loop. If they grasp the end of the rope at the wrong angle, it is usually not possible to successfully insert the rope correctly through the loop. After the initial warmup they performed five consecutive Rope Race tasks followed by three Peg Picker tasks. In the Peg Picker experiment, the test subject picks up small pegs with an alternative right or left instrument, transfers the peg to the other instrument, and then places the peg on a small spike. A total of 12 pegs should be put down correctly to complete the experiment (Fig. 3). The Peg Picker task is difficult, but the way in which you grasp the Peg is somewhat more forgiving compared to the exact precision of the grasping of the rope end required in the Rope Race task. After completion of the tasks, all subjects answered a follow-up questionnaire regarding their experience with the simulation training.

The Simball Box is a computer-based laparoscopic simulator [38], without any virtual reality software and has previously been used and described by our research group [39]. The advantage of this relatively low-cost simulator is that the clinic´s surgical instruments can be used with the simulator. All performance results of both TM and CG were saved on the computer hard drive of the Simball Box simulator, and the three last consecutive tasks were also displayed to the trainee as feedback on the Simball Box display. Video-screen recordings of both the TM and CG groups could not be recorded by the simulator but were saved and stored on the remote site for later analysis.

### Evaluation of data

The raw data regarding the objective simulator parameters were extracted from the simulator after the experiments and analysed with the statistical software JMP^®^ Pro 16.0.0 (SAS Institute Inc, Cary, NC, USA). The collected data regarding perceived emotions before and after the experiments were obtained from the questionnaires for which the students used a visual analogue scale (VAS) to express their emotions that they had before and after completing the experiments. The unidentified data were stored on a LaCie^®^ (Seagate Technology Holdings, Fremont, California, USA) 2Big Dock Thunderbolt 3 with RAID configuration in a locked room. The questionnaires and signed informed consents were archived at Sunderby Hospital in a folder and kept in a locked room.

### Statistical analysis

Statistical comparisons to identify differences between the nominal variables given in Table 1 between the two independent groups Tele-mentoring and Controls were done using the Pearson Chi-square test. For numeric data in Table 1, the Wilcoxon/Kruskal-Wallis tests were used. Intragroup comparisons between pre- and post-experiment values within each respective group given in Table 2 were statistically analysed using Matched Pairs analysis. In Table 3 the Matched Pairs analysis was used comparing the intragroup outcome of Rope Race 1 vs. Rope Race 5 and Peg Picker 1 vs. Peg Picker 3, respectively. A p-value < 0.05 was considered statistically significant. Statistical analysis was carried out using JMP® Pro version 16.0.0 (SAS Institute Inc., Cary, NC, USA).

**Table 3:** Simball Box results (mean ± standard deviation)

	Tele-mentoring					
	Rope Race 1	Rope Race 5	*P* (matched pairs)	Peg Picker 1	Peg Picker 3	*P* (matched pairs)
Overall score (%)	30.8 ± 12.1	43.4 ± 16.8	**0.004**	53.6 ± 10.3	65.8 ± 12.4	**0.019**
Total time (s)	224 ± 34	156 ± 29	0.058	360 ± 87	277 ± 44	**0.006**
Distance (cm)	797 ± 389	485 ± 122	**0.015**	960 ± 250	767 ± 149	**0.023**
Overall average speed (mm/s)	17.6 ± 2.2	15.5 ± 1.8	**0.001**	13.5 ± 1.7	14.0 ± 1.7	0.142
Average acceleration (mm/s^2^)	492 ± 53	428 ± 46	**0.001**	365 ± 44	374 ± 42	0.351
	**Control Group**					
	**Rope Race 1**	**Rope Race 5**	***P*** **(matched pairs)**	**Peg Picker 1**	**Peg Picker 3**	***P*** **(matched pairs)**
Overall score (%)	40.0 ± 11.5	50.6 ± 16.9	0.082	57.5 ± 13.7	70.8 ± 11.0	**0.007**
Total time (s)	176 ± 41	146 ± 53	0.187	334 ± 112	248 ± 42	**0.002**
Distance (cm)	547 ± 138	428 ± 140	0.098	899 ± 229	713 ± 90	**0.014**
Overall average speed (mm/s)	15.6 ± 2.2	15.0 ± 2.7	0.166	13.8 ± 1.8	14.6 ± 1.9	**0.009**
Average acceleration (mm/s^2^)	435 ± 62	423 ± 67	0.354	374 ± 41	394 ± 49	**0.027**

*P* < 0.05 statistically significant

## Results

### Participants

**Fig. 4:**
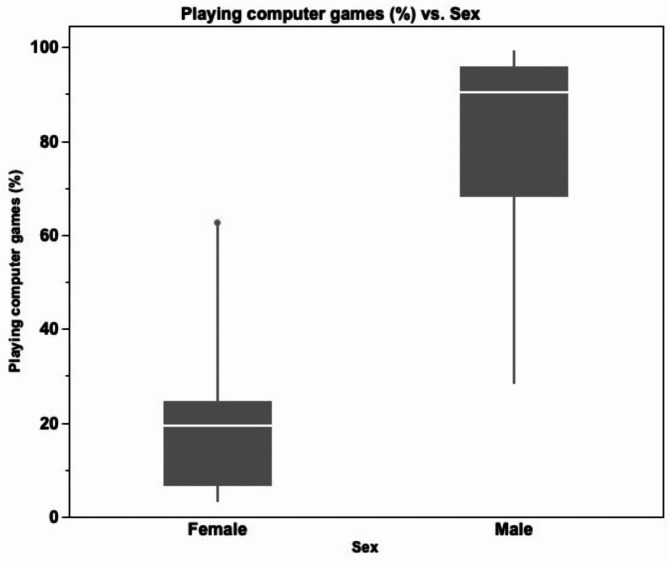
Computer gaming experience among females and males. Horizontal bars, boxes and whiskers represent the median, interquartile range, and range, respectively

The study participants of this study were randomized to either TM or CG. However, they were not stratified according to sex nor computer gaming experience, which unfortunately created some imbalance between the groups with a statistically significant difference regarding computer gaming experience in which the participants of the TM group had significantly more computer gaming experience, probably reflecting the male dominance of this group (Table 1; Fig. 4).

### Outcome data

#### Pre-experiment vs. post-experiment emotions

The emotions that the students experienced before and after the experiments in each group are reported in Table 2. The participants of both groups found that they liked the training more than they had expected with an increase in the TM group of 17.9% (*P* = 0.0002) and 13.8% (*P* = 0.001) for the CG (Table 2). No statistical differences regarding of how difficult the participants experienced the training compared to their pre-experiment expectations were found (Table 2).

#### Simball box results

In the TM group, the “overall score” of Rope Race improved significantly between Rope Race 1 and Rope Race 5 (30.8 ± 12.1 and 43.4 ± 16.8, respectively; *P* = 0.004), whereas this variable did not improve in CG (Table 3). Actually, the parameters´ “overall score”, “distance”, “overall average speed”, and “average acceleration” all improved significantly between Rope Race 1 and Rope Race 5 in the TM-group. The remaining variable in Table 3, “total time”, showed a trend to decrease (224 ± 34 to 156 ± 29; *P* = 0.058). Notably, none of the above-mentioned parameters in the CG group changed significantly during the Rope Race training. Of the five above mentioned parameters, “overall score”, “total time”, and “distance” were considered the most important ones for predicting the performance, whereas “overall averaged speed” and “average acceleration” we judged to be of less importance. In the TM group “overall score” of Rope Race increased 40.9%, “total time” was reduced by 30.3%, and finally “distance” was reduced by 39.1%. The corresponding values of the CG were 26.5%, 17.0%, and 21.8%, respectively.

In the Peg Picker procedures, all results given in Table 3 improved significantly between Peg Picker 1 and Peg Picker 3 in the CG, whereas only “overall score”, “total time”, and “distance” changed significantly in the TM group. However, of the three variables that changed, the magnitude of change was quite similar between the groups, namely, TM versus CG: (1) “overall score” (22.8% versus 23.1%), (2) “total time” (23.1% versus 25.7%), and (3) “distance” (20.1% versus 20.7%).

## Discussion

The purpose of the study was to analyse if it was possible to establish simulator teaching over a large geographical distance (900 km) and if this type of training was well received by the medical students. Furthermore, it was evaluated if TM objectively improved the outcome of the simulator exercises.

The present study provides evidence that it is possible to administer simulator training of basic surgical skills over large distances, which is well received by students (Table 2). Furthermore, analysis of the Simball Box data shows that TM significantly improved the outcome of the Rope Race task compared to no significant improvement in the CG group (Table 3). In the Peg Picker task both groups improved regarding overall score, total time, and distance. In addition, overall average speed and average acceleration improved in the GG group but not in the TM group (Table 3). Over the reasons for these differences one can only speculate but one possible explanation could be that those who received tele-mentoring reached their maximum performance level faster. Another possible explanation to these results might also be that the Rope Race task is more visuospatially demanding and therefore gain more advantage from the given TM.

Moreover, this study confirms the still present male dominance regarding computer gaming experience (Fig. 4). The gender difference seen in gaming experience in this study is in line with previous studies published by our group [40]. As mentioned previously, surgical laparoscopic simulators have been used to enhance proficiency and provide positive effects on the surgical outcomes [2–4]. Although, several potentially available and financially feasible simulators have been designed for home-training [10], not many are being used [12]. In a systematic review of voluntary participation in simulation-based laparoscopic skills training, Gostlow et al. found that it is important to create intrinsic or extrinsic motivating factors in conjunction with free time to successfully increase the rate of voluntary simulator training [17]. Additionally, instructor feedback has been shown to enhance simulator performance in several previous studies [14, 15, 41].

Furthermore, the use and incorporation of telehealth and video conferences skyrocketed during the evolve of the Covid-19 pandemic [22]. For a large group of workers, homes turned into office spaces [42]. Unfortunately, the same cannot be said about surgeons. A study in the United Kingdom (UK) described a negative impact on the clinical surgical training for surgeons [27]. A recent study from South Africa also highlights the impact that the Covid-19 lockdown had in terms of increased morbidity and mortality due to insufficient surgical practices [24].

Therefore, in this study, the positive effects that TM could have in facilitating basic surgical skills simulator training among medical students are suggested.

### Strengths and limitations

One obvious limitation of this study is that the study group was composed of medical students and not surgical residents. Thus, whether the results are transferable between these groups remain unanswered. Second, the amount of time to practice may be an additional limitation with respect to how the subjects would perceive their training, regardless of the presence of a supervisor or not. Previous studies, however, have shown that only a few trials are needed to reach an acceptable level of proficiency [43, 44]. Third, prior to the study, no power calculation was performed. However, our study with 20 participants exceeded earlier and frequently cited randomized controlled studies within this field that, at most, involved no more than 16 participants (Seymour et al. 2002, N = 16; Grantcharov et al. 2004, N = 16; Ahlberg et al. 2007, N = 13) [2–4]. Nevertheless, the benefit of a power calculation prior to the study is reasonable, especially when performing subgroup analyses. The randomization divided the participants into two groups with 10 participants in each group. Only medical students were enrolled, but unfortunately due to the lack of gender stratification, with more male subjects ending up in the TM group. Moreover, prior video-gaming experience was also more frequent in the TM group compared with the CG. Males, rather than females, presented a higher level of computer gaming experience (Fig. 4). By including a larger number of participants and using block randomization, the risk of a selection bias would have been covered. Also, the fact that there was only one mentor that conducted the mentoring in the study could have impacted the outcome. However, since this was a rather small study with only 10 students getting TM, we believe that having only one mentor improved the continuity of the study. In future studies with more participants, however, we believe it may be beneficial to include additional mentors to reduce the importance of the mentors’ skill on the outcome. Furthermore, when working with technical and online solutions, the risk of internet-connection and electrical/technical failures exist, which could jeopardize and/or delay training including any attempts of feedback. Finally, the limited number of participants is something that can make statistical calculations somewhat uncertain, but since we used matched pair analyses where each person is his or her own control, this uncertainty is compensated for to a certain degree. Also, the issue of financing TM teaching is an important point that has not been touched upon and where there can be big differences both between different countries and healthcare systems.

## Conclusions

Our study indicates that overall, tele-mentoring was well received by the students. Furthermore, our limited study suggests that TM had a positive impact on the presumably more complex Rope Race simulation task in the laparoscopic simulator, Simball Box. We suggest that surgical simulator tele-mentoring over long distances could be a viable way to both motivate and increase laparoscopic basic skills training in the future. Further studies with more participants, preferably surgical residents, and mentors are planned.

### Electronic supplementary material

Below is the link to the electronic supplementary material.

Supplementary Material 1

Supplementary Material 2

## Data Availability

The datasets used and/or analysed during the current study are available from the corresponding author on reasonable request.

## List of Abbreviations

Apple: Apple Inc., Cupertino, California, USA
ASUS: Asustek, Taipei, Taiwan
CG: Control group
Intel: Intel Corp., California, USA
JMP Pro: Statistical software by SAS Institute inc., Cary, NC, USA
LaCie: Seagate Technology Holdings, Fremont, California, USA
Logitech: Logitech International SA, Lausanne, Switzerland
OR: Operating room
Peg Picker: A procedure in the Simball Box simulator where the test person shall pick up small pegs with an alternative right or left instrument, transfer the peg to the other instrument, and then place the peg on a small spike
RAID: Random Array of Independent Disks
Rope: Race A procedure in the Simball Box simulator where the test person shall thread a thin rope through eight loops
Samsung: Samsung Electronics, Republic of Korea
Simball Box: Laparoscopic simulator by Surgical Science Sweden AB, Gothenburg, Sweden
TM: Tele-guided mentoring
VAS: Visual analogue scale
Zoom: Zoom Video Communications Inc., San Jose, California, USA
