# Supplementary material for: Tele-mentoring - a way to expand laparoscopic simulator training for medical students over large distances: a prospective randomized pilot study
Source: BMC Med Educ. 2023 Oct 10;23:749. doi: 10.1186/s12909-023-04719-x (PMC10566045; doi:10.1186/s12909-023-04719-x)
Supplement: Supplementary file 2 — Supplementary Material 2 [file 12909_2023_4719_MOESM2_ESM.doc]

**BMC Medical Education**

**COVER LETTER**

Dear Editor-in-Chief,

We have now read through the views from reviewer 1 and changed the manuscript in accordance with the reviewer's suggestions in the conclusion. We now hope that the manuscript meets the standards that BMC Medical Education requires for the manuscript to be accepted.

Stockholm 23-09-11

Sincerely yours,

Lars Enochsson, MD, PhD

Professor of Surgery,
Department of Surgical and Perioperative Sciences,

Division of Surgery, Umeå University, Umeå, Sweden

And Affiliated to CLINTEC, Karolinska Institutet, Stockholm, Sweden
E-mail:	[lars.enochsson@umu.se](mailto:lars.enochsson@umu.se)
Phone:	+46736260800

**COVER LETTER**

Dear Editor-in-Chief,

We very much appreciated the review of our manuscript that was done. We have now done a major revision in accordance with many of the comments made by the reviewers and think it has improved the manuscript significantly. We now hope that it is up to the standards necessary for acceptance and publication.

Stockholm 23-07-27

Sincerely yours,

Lars Enochsson, MD, PhD

Professor of Surgery,
Department of Surgical and Perioperative Sciences,

Division of Surgery, Umeå University, Umeå, Sweden

And Affiliated to CLINTEC, Karolinska Institutet, Stockholm, Sweden
E-mail:	[lars.enochsson@umu.se](mailto:lars.enochsson@umu.se)
Phone:	+46736260800

Dear Editor-in-Chief,

Please find enclosed our manuscript **“Video-guided Tutoring – a way to expand laparoscopic simulator training over large distances: a prospective randomized pilot study”** by Oussi et al., which we would like to submit for publication as an original research article in BMC Medical Education.

Our manuscript has been language reviewed by American Manuscript Editors, project nr 90008.

We believe our findings would appeal to the readership of the journal, with findings describing the benefits of video-guided tutoring feedback using Zoom over distances in laparoscopic simulation training, something to consider when preparing a training curriculum.

We confirm that this manuscript has not been published elsewhere and is not under consideration by another journal.

All authors have nothing to disclose, approved of the manuscript and agree with its submission to BMC Medical Education.

We look forward to hearing from you at your earliest convenience.

Stockholm 23-01-09

Sincerely yours,

Lars Enochsson, MD, PhD

Professor of Surgery,
Department of Surgical and Perioperative Sciences,

Division of Surgery, Umeå University, Umeå, Sweden

And Affiliated to CLINTEC, Karolinska Institutet, Stockholm, Sweden
E-mail:	[lars.enochsson@umu.se](mailto:lars.enochsson@umu.se)
Phone:	+46736260800
